# CircRNA在肺癌诊断与发生发展及耐药中的作用进展

**DOI:** 10.3779/j.issn.1009-3419.2019.08.09

**Published:** 2019-08-20

**Authors:** 天翔 陈, 运海 杨

**Affiliations:** 100021 北京，国家癌症中心，国家肿瘤临床医学研究中心，中国医学科学院北京协和医学院肿瘤医院胸外科 Shanghai Lung Cancer Center, Shanghai Chest Hospital, Shanghai Jiao Tong University, Shanghai 200030, China

**Keywords:** CircRNA, MiRNA海绵, 肺肿瘤, CircRNA, MicroRNA sponge, Lung neoplasms

## Abstract

肺癌在全球范围内的致死率一直居高不下。近年来针对多种分子分型的靶向药物已成为中晚期肺癌治疗的新有效手段，但是肺癌在早期诊断以及长期有效的治疗上仍然面临着严峻的挑战。环状RNA（circular RNA, circRNA）是一类具有环形结构的独特RNA分子，具有优异的稳定性以及表达特异性。越来越多的研究发现circRNA在肿瘤中表达异常，这种异常表达不仅与肿瘤的恶性相关，同时可以参与调控肿瘤进展，为肿瘤的诊断与治疗提供了新的思路。因此，本文就circRNA在肺癌中的表达、诊断、预后价值以及发生发展机制展开综述，以期为肺癌的早期诊断与治疗寻找新的靶点。

## Abstract (en)

The mortality rate of lung cancer has been high all over the world. In recent years, targeted drugs have become a new and effective method for the treatment of advanced lung cancer, but the early diagnosis and long-term effective treatment are still great challenges in lung cancer. Circular RNA (circRNA) is a unique RNA molecule with ring structure, which exhibits excellent stability and expression specificity. Increasing studies found that the expression of several circRNAs in tumors were abnormal. This abnormal expression is not only related to malignancy of tumors, but also involved in regulating the progress of tumors, which provides a new way for the diagnosis and treatment of tumors. Therefore, this article reviews the expression, the diagnostic and prognostic value as well as pathogenesis of circRNAs in lung cancer in order to find new targets for early diagnosis and treatment of lung cancer.

肺癌是我国乃至世界发病率及病死率最高的恶性肿瘤之一^[1]^。大约85%的肺癌是非小细胞肺癌（non-small cell lung cancer, NSCLC），早期NSCLC经手术治疗5年生存率可达70%-90%^[2]^，但是约75%的患者在初诊时已是进展期或晚期^[3]^。小细胞肺癌（small cell lung cancer, SCLC）在肺癌中仅占15%左右，但是SCLC更具侵袭性，超过90%的患者在诊断时已是晚期^[3]^，5年生存率仅为5%。晚期肺癌是肺癌治疗的主要挑战，近年来以表皮生长因子受体（epidermal growth factor receptor, EGFR）、间变性淋巴瘤激酶（anaplastic lymphoma kinase, ALK）、ROS1等为靶点的药物均临床上展现了卓越疗效，已成为仅次于化疗的主要治疗方式。例如，三代EGFR抑制剂奥西替尼（AZD9291）的中位无进展生存期（progression-free survival, PFS）可达19.3个月^[4]^，二代ALK抑制剂色瑞替尼在无脑转移患者中的PFS可超过2年^[5]^。但是这些抑制剂仅适用于具备该靶点的患者，而其他患者则无法从中获益，只能选择收效甚微的化疗。因此深入研究肺癌发生发展的机制，寻找新的靶点刻不容缓。环状RNA（circular RNA, circRNA）是一类缺少5’与3’端，由共价键形成闭合环状的特殊RNA。这种特殊的环状结构使得circRNA对核酸外切酶RNase R耐受，在胞内的半衰期超过48 h，呈现出优异的稳定性^[6]^。此外circRNA在真核生物内含量丰富，表达具有组织特异性与疾病特异性，同时参与调控肿瘤的发生与转移，有望成为肿瘤的生物标记物与治疗靶点。

## 1 CircRNA在肺癌中的表达及意义

### 1.1 CircRNA的诊断价值

提高早期诊断率是改善肺癌生存率的关键。目前关于肺癌分子标记物的研究主要集中在肿瘤循环细胞、循环DNA、循环miRNA、血清肿瘤标志物、肺癌自身抗体等，前三种标记物在早期肺癌的血液含量极为有限，敏感性不足，后两种标记物的敏感性与特异性均不理想^[7]^，而circRNA的出现则为肺癌的早期诊断带来了新的希望。Zhu等^[8]^利用circRNA芯片技术在肺腺癌患者肿瘤组织中鉴定出39个异常表达的circRNA，经验证后发现hsa_circ_0013958在细胞株、组织及血浆中均高表达。且发现组织hsa_circ_0013958的表达与原发灶-淋巴结-远处转移（tumor-node-metastasis, TNM）分期、淋巴结转移相关。以健康人群为对照，血浆与组织hsa_circ_0013958的受试者操作特征曲线（receiver operating characteristic curve, ROC）曲线下面积（area under the curve, AUC）分别为0.794与0.815。随后研究人员对hsa_circ_0013958在不同分期肺腺癌的AUC分别进行了评价，发现组织hsa_circ_0013958在早期肺腺癌的诊断中依旧表现优良，其在Ⅰ期与Ⅱ期的AUC依次为0.75、0.766。Hang等^[9]^利用RNA-seq结合qRT-PCR验证，发现circFARSA在NSCLC患者血浆中表达上调，并与肿瘤组织中circFARSA的表达呈线性相关，而FARSA的mRNA在血浆中无法检测出。以健康人群为对照，血浆circFARSA具有一定的诊断价值，AUC为0.71。CircRNA_102231与hsa_circ_0000729在肺腺癌组织中均异常高表达，并与TNM分期、转移相关，circRNA_102231的AUC为0.897，敏感性与特异性分别为0.812、0.887，hsa_circ_0000729的AUC为0.815^[10, 11]^。此外，Tan等^[12]^发现*EML4-ALK*融合可表达融合circRNA（F-circEA），而F-circEA仅特异性地表达于EML4-ALK阳性患者的血浆内，提示F-circEA有望开发为EML4-ALK阳性的诊断标记物。

### 1.2 CircRNA的预后价值

预后评价是制定临床方案的重要参考项，对于延长患者生存期意义重大。Zou等^[13]^发现circ-0067934在NSCLC肿瘤组织及细胞株内均显著高表达。*Kaplan-Meier*生存与对数秩检验分析表明circ-0067934高表达与总体生存期负相关，*Cox*比例风险分析指出circ-0067934可作为NSCLC患者不良预后判断的独立风险因子。CiRS-7作为超级海绵，在肺癌中的预后价值也有一定的研究。Su等^[14]^分别检测了128例NSCLC患者肿瘤组织标本发现ciRS-7异常上调，miR-7异常下调。而高表达ciRS-7、低表达miR-7的NSCLC患者往往较为晚期，一般瘤体较大，伴有淋巴结转移，生存期较短，作者认为高表达ciRS-7与低表达miR-7均是患者不良预后的独立预测因子。Liu等^[15]^发现circ_0001649在NSCLC肿瘤组织中表达下调，其表达与TNM分期、淋巴结转移、预后相关。Hsa_circRNA_000122在肺鳞癌中表达下调，低表达hsa_circRNA_000122的患者总体生存期显著缩短，有望开发为肺鳞癌的预后标记物^[16]^。

## 2 CircRNA在肺癌进展中的作用及机制

与大多数肿瘤一样，肺癌在发生发展中亦表现出恶性增殖、凋亡抵制、侵袭增强以及无法避免的耐药性。CircRNA虽然在肺癌中异常表达，并与TNM分期、淋巴结转移相关，但是circRNA是否参与了肺癌的发生发展呢？

### 2.1 促进肺癌增殖

Hsa_circ_0013958在肺腺癌中异常高表达，敲除hsa_circ_0013958可以抑制细胞的增殖^[8]^。Zhu等^[17]^首先利用原位杂交对hsa_circ_0013958进行定位分析，发现其主要分布在胞浆，作者推测其可能作为miRNA海绵进而促进增殖。随后利用CIRCBASE以及STARBASE数据库对hsa_circ_0013958可能结合的miRNA进行预测。使用荧光素酶报告基因系统进一步验证，鉴定出了miR-134-5p。此外，过表达hsa_circ_0013958可以增加细胞周期D1的表达，而同时过表达miR-134可抑制其表达，提示hsa_circ_0013958可作为miR-134的海绵，上调细胞周期D1的表达进而促进细胞增殖。CircPVT1在NSCLC患者的组织及血清中均过度表达，敲除circPVT1可以抑制细胞增殖、阻滞细胞周期于G_0_期/G_1_期。通路报告芯片检测发现敲除circPVT1后，E2F通路发生了最显著的抑制，同时过表达E2F2可以逆转circPVT1沉默后的细胞生存抑制。E2F2是E2F转录因子家族的一员，可以通过启动子区的E2识别位点与DPDP1多肽共结合于DNA，推进包括肺癌在内的多种肿瘤的细胞周期、促进其恶性增殖。数据库预测及荧光素酶报告系统证明miR-125b与circPVT1、E2F2发生靶向结合，沉默circPVT1将促进miR-125b与E2F2的结合，抑制NSCLC细胞增殖。在体内敲除circPVT1抑制了瘤体的生长与转移，以上均提示circPVT1有望开发为NSCLC治疗的靶点。此外，Chen等^[18]^发现hsa_circ_100395通过miR-1228/TCF21信号轴推进细胞周期、促进肺癌细胞增殖。Circ-PUM1可作为miR-326的海绵，增加细胞周期D1的表达，促进肺腺癌细胞的增殖^[19]^。

### 2.2 抑制肺癌凋亡

Yu等^[20]^发现circHIPK3在肺癌细胞中表达增加，沉默circHIPK3显著抑制细胞的生存，并诱导其凋亡。为找到circHIPK3在肺癌中可能结合的miRNA，Yu等^[20]^在沉默了circHIPK3的细胞中分别干扰三个备选miRNA，发现仅有转染了miR-124抑制剂的细胞发生了表型的逆转。此外，沉默circHIPK3可抑制miR-124靶基因*SphK1*、*STAT3*及*CDK4*的表达，抑制miR-124可增加这些靶基因的表达，提示circHIPK3通过miR-124-SphK1/STAT3/CDK4信号轴传递凋亡抵制信号，从而促进肺癌的恶性进展，为NSCLC的治疗提供了新的策略。CircUBAP2与hsa_circ_0000064在肺癌组织中均表达增加，分别沉默这两种circRNA后均可以抑制细胞增殖、促进凋亡^[21, 22]^。沉默circUBAP2可以抑制Bcl-2、Survivin的表达，并上调Bax的表达，同时JNK、ERK1/2的活性也被抑制，提示circUBAP2可能通过干预JNK与ERK1/2通路促进细胞的凋亡。荧光素酶报告基因分析miR-339-5p、miR-96-3p与miR-135b-3p均可以与circUBAP2结合，但这些miRNA是否可以调控circUBAP2抑制凋亡的作用并不清楚。沉默hsa_circ_0000064后，Caspase-3、Caspase-9、Bax表达增加，bcl-2表达减少，关于hsa_circ_0000064与凋亡抑制之间的信号转导需要更多的研究。

### 2.3 促进肺癌的转移

肿瘤转移是造成癌症患者死亡的最主要原因之一。上皮-间质转化（epithelial-mesenchymal transition, EMT）被认为与肿瘤细胞的侵袭转移的相关。目前发现NSCLC高表达TGF-β可以刺激NSCLC细胞自身的EMT。TIF1γ在NSCLC中低表达，敲除或下调TIF1γ可以增强TGF-β诱导的EMT作用。Wang等^[23]^首先在细胞中过表达TGF-β诱导EMT发生，然后进行circRNA芯片分析，并检测发现TIF1γ表达减少。于是假设某些circRNA的异常表达抑制了TIF1γ表达，进而激活了TGF-β诱导的EMT。根据算法对TargetScan/miRBase以及miRanda数据库分析后发现，circPTK2与TIF1γ均存在miR-429/miR-200b-3p的结合位点。荧光素酶报告基因系统证明circPTK2与TIF1γ均可与miR-429/miR-200b-3p的直接结合。该研究发现circPTK2可以通过海绵miR-429/miR-200b-3p抑制TIF1γ的表达来调控NSCLC的转移，丰富了TGF-β诱导的EMT机理。Qu等^[24]^发现hsa_circ_0020123在NSCLC中表达增加，并且与淋巴结转移、TNM分期显著相关，敲除hsa_circ_0020123可以抑制肿瘤的侵袭及转移，过表达hsa_circ_0020123则表现出相反的表型。数据库预测及荧光素酶报告基因系统证明发现hsa_circ_0020123可以通过miR-144-ZEB1/EZH2轴促进肿瘤的转移。ZEB1是激活EMT的重要转录因子，EZH2是Zeste同源增强子，可激活Notch1促进肿瘤的EMT^[25]^。虽然该研究并没有检测EMT的标志物，但是推测hsa_circ_0020123可能通过miR-144-ZEB1/EZH2轴启动NSCLC的EMT、促进其转移。此外，circ-0067934、hsa_circ_0079530及hsa_circ_0007534在NSCLC患者中均表达增加，与淋巴结转移相关。分别沉默这些circRNA均可削弱细胞的迁移与侵袭能力、逆转EMT表型，表明这三种circRNA均可通过调控EMT促进NSCLC的转移^[13, 26, 27]^。

### 2.4 诱导肺癌耐药

虽然现有靶向药物在肺癌的治疗中表现良好，但是每一种药物均会迎来耐药，使得晚期肺癌患者陷入了无药可用的绝境，因此深入理解耐药机制、寻找新的靶点异常重要。Xu等^[28]^利用高通量circRNA芯片检测了A549敏感株及其紫杉醇耐药株A549/Taxol。与敏感株相比，A549/Taxol中2, 909个circRNA表达显著上调，8372个circRNA显著下调，提示circRNA的异常可能参与调控了紫杉醇耐药的发生。Zhou等^[29]^利用circRNA芯片在HCC827敏感株及其埃克替尼耐药株HCC827I/R中筛选出了hsa_circ_0004015。临床样本分析表明hsa_circ_0004015在NSCLC患者组织中表达增加，与不良生存率相关。在HCC827中过表达hsa_circ_0004015可诱导细胞对吉非替尼的耐受，在HCC827I/R中敲除hsa_circ_0004015后可增加HCC827I/R对吉非替尼的敏感性。进一步研究发现hsa_circ_0004015可以通过miR-1183/PDPK1轴调控细胞的耐受性。

## 3 讨论与展望

部分circRNA在肺癌中稳定异常表达，具有良好的灵敏度与特异性，并被证实与不良预后相关，为肺癌的早期诊断与预后标记物的开发提供了新的选择。随着时间的推移，将会有更多具有临床价值的circRNA被发现，但是现有研究存在一些局限性：①研究局限于NSCLC，而circRNA在SCLC患者中的诊断/预后价值研究尚属空白。临床研究发现，Ⅰ期SCLC患者经手术切除辅助化疗后5年生存率可达52%^[30]^，约是晚期患者5年生存率的10倍，因此通过探索SCLC中潜在circRNA标志物是提升早期诊断率的重要途径。②主要评估了肺癌组织circRNA的表达，而血液circRNA的表达与诊断价值尚不清楚。③多数研究是基于单中心、小样本的单个circRNA研究。肺癌是一种高度异质性的肿瘤，现有的研究结果都是建立在单中心、小样本数据基础上，因此这些发现的circRNA恐怕未必能够在多中心、大样本数据中得到理想验证，故针对多个circRNA构建肺癌相关的circRNA组（circRNA panel）进行研究才有机会推动circRNA真正走向临床。

胞浆circRNA通过海绵miRNA调控肺癌的发生发展是目前机制研究的主流，而核内circRNA在肺癌中的机制研究尚不清楚。然而大多数circRNA并不具备丰富的miRNA结合位点，对大量circRNA作为miRNA海绵提出了质疑，同时提醒我们circRNA在肺癌中的作用机制可能不仅局限于miRNA的海绵^[31]^。翻译蛋白质，尤其是具有活性的蛋白质，均是circRNA的重要功能，而肺癌中异常表达的circRNA或许可以通过翻译出活性蛋白质调控其恶性进展，这些活性蛋白是否可以成为新的靶点是未来研究一个重要方向。此外，在circRNA调控肺癌进展的机制研究中，均以单一的肿瘤细胞为对象，而肿瘤微环境中的circRNA是否异常表达，微环境中不同细胞中的circRNA是否异常表达，以及这些circRNA是否可以在不同细胞中传递进而促进肿瘤进展均不清楚。肿瘤微环境是肿瘤发生发展的土壤，深入理解circRNA在肿瘤微环境中的作用机制，在肿瘤早期未成熟前进行干预是重要的研究方向。

现有研究提示未来circRNA在肺癌的临床诊断，治疗及预后预测中的潜在作用，而circRNA在肺癌发生发展中的已知作用机制将是研究肺癌新治疗方法的潜在靶点。
